# Neuronal surface autoantibodies in dementia: a systematic review and meta-analysis

**DOI:** 10.1007/s00415-020-09825-0

**Published:** 2020-04-18

**Authors:** Lucy L. Gibson, Anna McKeever, Alexis E. Cullen, Timothy R. Nicholson, Dag Aarsland, Michael S. Zandi, Thomas A. Pollak

**Affiliations:** 1 Department of Old Age Psychiatry, Institute of Psychiatry, Psychology and Neuroscience, King’s College London, London, UK; 2 School of Clinical Medicine, Addenbrooke’s Hospital, University of Cambridge, Hills Rd, Cambridge, CB2 0SP UK; 3 Department of Psychosis Studies, Institute of Psychiatry, Psychology and Neuroscience, King’s College London, London, UK; 4 Centre for Age-Related Diseases, Stavanger University Hospital, Stavanger, Norway; 5 UCL Queen Square Institute of Neurology, National Hospital for Neurology and Neurosurgery, Queen Square, London, WC1N 3BG UK

**Keywords:** Autoimmune dementia, Atypical dementia, NMDAR antibody, Antibodies in dementia

## Abstract

**Introduction:**

Neuronal antibodies can cause encephalopathy syndromes often presenting with subacute cognitive impairment, sometimes resembling neurodegenerative dementias.

**Methods:**

We searched Medline and Embase for studies reporting associations between neuronal surface antibodies in all-cause dementia versus controls. Random-effects meta-analysis was used to pool adjusted estimates across studies.

**Results:**

Six studies were included, all reporting frequency of serum NMDAR antibodies in dementia with four also reporting frequency in atypical dementias. Both IgG [OR = 8.09 (1.51; 56.85), *p* = 0.036] and IgA/IgM NMDAR antibodies [OR = 42.48 (11.39; 158.52), *p* < 0.001] were associated with atypical dementia, but neither were associated with all-cause dementia.

**Discussion:**

In the first meta-analysis to explore this literature, serum IgG and IgA/IgM NMDAR antibodies were significantly more common in atypical dementias. However, methodological issues and small-sample sizes necessitate caution interpreting this result. Further studies measuring both serum and CSF antibodies are needed to investigate the role of neuronal antibodies in dementia, since evidence of pathogenicity in even a subset of patients could pave the way for novel treatment options.

## Introduction

The last decade has witnessed a rapid expansion in the description of central nervous system diseases associated with autoantibodies targeting neuronal and glial cell surface antigens. While the cascade of downstream effects has yet to be fully elucidated for many such autoantibodies, it is likely that their antigen-specificity determines their functional or pathogenic effects [1]. Autoimmune encephalitis denotes a group of central nervous system disorders associated with brain-directed autoantibodies with characteristic clinical syndromes. Most common is N-methyl-D-aspartate receptor (NMDAR) antibody encephalitis, in which IgG antibodies to the NR1 subunit of the NMDAR cause receptor internalisation, dynamic disorganisation of the NMDAR at the synapse, and reduced NMDAR-related excitatory post-synaptic potentials [2–4]. The associated syndrome is well described, featuring prominent neuropsychiatric symptoms and cognitive impairment with memory, attentional, and executive deficits evident alongside movement disorder, seizures, and dysautonomia [5]. Cognitive impairment is a central feature of most of the autoimmune encephalitides though variation is seen in the affected cognitive domains. In LGI1 and CASPR2 encephalitis, antibodies bind to accessory proteins on voltage-gated potassium channels, typically causing disorientation, confusion, and impairments in autobiographical memory [6]. In NMDAR encephalitis, cognitive deficits can persist for years after the initial insult and, in the acute phase, are often more pronounced in older adults [7, 8].

Neuronal surface autoantibodies have also been detected in patient populations outside the context of encephalitis, and particularly for IgA/IgM NMDAR antibodies, seroprevalence appears to be greater in older populations, although the clinical consequences of this are not yet known [9, 10]. The clinical profile of antibody-mediated cognitive impairment has led to particular interest in the potential pathogenicity of neuronal autoantibodies in neurodegenerative dementias. This is especially pertinent given the increasing evidence supporting an autoimmune aetiology in many neurodegenerative dementias: Alzheimer’s disease is significantly more common in most types of autoimmune disease, and genome-wide association studies have demonstrated numerous shared loci between Parkinson’s disease and autoimmunity [11, 12]. Furthermore, functional autoantibodies have recently been suggested to play a causative role in the pathogenesis of Alzheimer’s disease [13]. Antibodies to a range of endogenous receptors have also been associated with increased mortality and neuropsychiatric symptoms in Alzheimer’s disease [14].

Given the evidence associating NMDAR hypofunction with cognitive decline in ageing, NMDAR antibodies could also be a promising focus [15]. Indeed, there are numerous cases of autoantibody-mediated, immunotherapy-responsive dementias which mimic primary neurodegenerative dementias [16–18]. Furthermore, IgA NMDAR antibodies have been associated with ‘atypical dementia’ in older adults, characterised by a slowly progressive cognitive impairment which is reversible with immunotherapy [19]. While all NMDAR antibody isotypes show potential pathogenicity in vitro [20], distinction must be made between the encephalitogenic IgG isotype and the IgA and IgM isotypes which are of uncertain clinical relevance, but have been associated with cognitive impairment outside the context of encephalitis [21]. In addition, IgLON5 antibodies are associated with neuronal accumulation of hyperphosphorylated tau concurrent with the neuronal loss and gliosis typical of neurodegenerative disease [22].

Recently, the term ‘autoimmune dementia’ has been proposed to describe patients with a subacute cognitive impairment which is reversible with immunotherapy [23]. While the prevalence of this condition is currently unclear, in a study of 56 patients initially diagnosed with a neurodegenerative or prion disorder, immunotherapy led to improvements across cognitive domains in a third of the patients [17]. This is of particular interest given the frustrating lack of progress that has been made to date with other disease modifying therapies in dementia. Indeed, identifying a pathogenic link between neuronal antibodies and cognitive decline in even a subset of patients with dementia could offer the potential of transformational new treatment options.

However, despite growing interest in the role of neuronal antibodies in dementia, to our knowledge, there have been no attempts to systematically appraise the extant literature. We therefore conducted a systematic review and meta-analysis which aimed to (1) systematically appraise studies comparing frequency of neuronal autoantibodies (NMDAR, AMPAR, GABA_A_R, GABA_B_R, GAD, LGI1, and CASPR2) in patients with a primary diagnosis of dementia with healthy controls and (2) determine the magnitude and consistency of effects using meta-analytic techniques.

## Methods

The protocol was registered on PROSPERO (registration number: CRD42018115200) and reported according to the *Meta-Analysis of Observational Studies in Epidemiology (*MOOSE) guidelines [24].

### Search strategy

Embase, Ovid MEDLINE^®^ and Epub Ahead of Print, In-Process & Other Non-Indexed Citations, Daily and Versions^®^ were searched for articles published up to July 2019. We used the search terms: (dementia OR alzheimer* OR fad OR lewy bod* OR frontotemporal OR ftd OR progressive supranuclear palsy OR psp OR primary progressive aphasia OR ppa OR vascular OR korsakoff* OR pick*) AND (antibod* OR auto-antibod* OR autoantibod*) AND (*n*-methyl-d-aspartate OR nmda OR nmdar OR ampa OR ampar OR glutamate OR glur OR gaba OR potassium channel OR caspr2 OR lgi1 OR vgk* OR dppx OR d2r). The search was restricted to studies published in English. We included studies published in peer reviewed journals, while conference reports were excluded. Bibliographies of studies and reviews were searched manually for further eligible articles.

### Study selection

Eligible articles were observational studies including at least ten participants with what we here denote a dementia syndrome: Alzheimer’s disease (AD) including familial Alzheimer’s disease (FAD), vascular dementia (VD), dementia with Lewy bodies (DLB), frontotemporal dementia (FTD) with primary progressive aphasia (PPA) and Pick’s disease inclusive, progressive supranuclear palsy (PSP), and Korsakoff’s syndrome, which performed assays for antibodies against anti-neuronal antigens and reported seropositivity or serum titres and included a healthy comparison group. The results were screened independently by two medically trained reviewers (A. McK and L.L.G.) who reached a consensus on potentially eligible articles. When study cohorts overlapped, only the larger was included.

### Data collection

Data were extracted independently by two reviewers (A. McK and L.L.G), who were not blinded to author names, journals, or institutions. Data extraction included: study recruitment procedure, description of participant groups and exclusion criteria, demographics, antibody assay methods (immunostaining methods, blinding, target antigen, antibody isotype, and seropositive threshold), and assay results.

### Assessment of studies

Study quality was assessed independently by two reviewers (A. McK and L.L.G), using an adapted version of the Newcastle Ottawa Scale (NOS), a quality assessment tool for case–control studies. Discrepancies in quality ratings were resolved by a third researcher (A.E.C). The NOS addresses eight areas including: participant selection, case definition, matching, and exposure. These were modified as appropriate for our analysis, giving ten modified items, which were rated for each study. Studies were scored 0 or 1 for eight of the ten items, and scored 0, 1, or 2 for two items, giving a maximum quality score of 12.

### Statistical analysis

Statistical analyses were conducted using Stata version 15. Meta-analyses were performed to compare antibody presence in each dementia group (all-cause, typical, and atypical) with healthy controls. As we suspected that the true effect might vary across studies (owing to differences in dementia groups examined), random-effects models were employed for all analyses with inverse weighting applied [25]. We had originally intended to include all neuronal autoantibodies in the analysis. However, given that the search only identified one study quantifying the prevalence of autoantibodies other than NMDAR antibodies in dementia syndromes, we subsequently restricted our analysis to NMDAR antibodies. For NMDAR antibodies, the two outcome measures were seropositivity for IgG NMDAR antibodies and separately for IgA/IgM NMDAR antibodies combined. These antibody isotypes were assessed separately on the basis that their respective clinical implications are suspected to differ [26]. Statistically significant heterogeneity was determined via the Cochran *Q* statistic, the *I*^2^ statistic (estimating the percentage of the variability in odds ratios due to heterogeneity) was also calculated and categorised as likely ‘unimportant’ (0–40%), ‘moderate’ (30–60%), ‘substantial’ (50–90%), or ‘considerable’ (75–100%) depending on the magnitude/direction of effects and statistical significance of heterogeneity [27]. Exploratory meta-regression analyses were also performed to examine the relationship between study quality (NOS score) and effect sizes.

## Results

### Search strategy

**Fig. 1:**
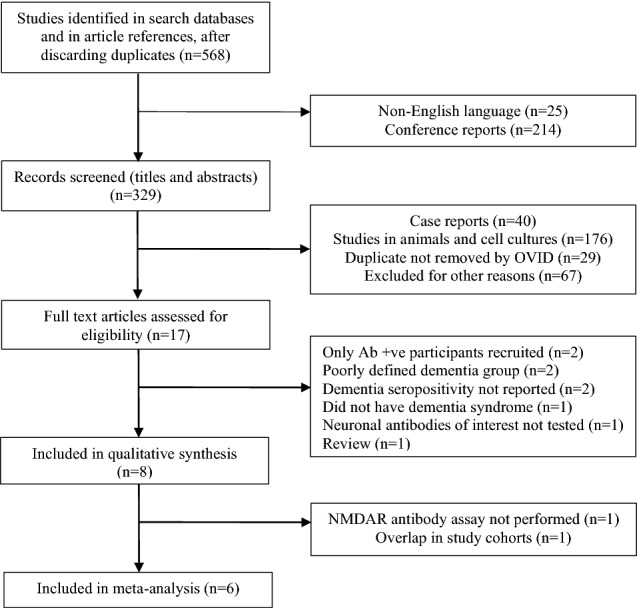
Search process

Once duplicates were removed, our search returned 568 results (see Fig. 1). After exclusion of conference reports and non-English language papers, abstracts were screened for 329 papers. Additional duplicates, animal studies and case reports were then excluded. For the remaining 17 studies, the full-text was assessed for eligibility. Nine further studies were then excluded due to inadequate definition of dementia syndromes, reporting exclusively seropositive patients, not reporting seropositivity in dementia patients or not testing the neuronal autoantibodies of interest.

Eight studies met the inclusion criteria [9, 19, 28–33]. However, one study [9] was subsequently excluded due to having an overlapping sample with a larger study [30]. A second study examined α-amino-3-hydroxy-5-methyl-4-isoxazolepropionic acid receptor (AMPAR) antibodies only [28] and so was excluded from the meta-analyses. Six remaining studies examined NMDAR antibodies and were included in the meta-analysis [19, 29–33].

### Study characteristics

#### Sample size, demographic factors, and group status

Across the six studies, we included 678 individuals with a typical dementia disease, including Alzheimer’s disease, frontotemporal dementia, vascular dementia, dementia with Lewy bodies, Parkinson’s disease dementia and progressive supranuclear palsy, 520 healthy controls, and 70 participants with an atypical dementia. Gender and age distribution were not reported in all studies. Demographic information is shown in Table 1.

**Table 1:** Demographics for patients and healthy controls from studies included in the meta-analysis

References	Category	Diagnosis	*n*	Serum NMDAR antibody positive (%)	CSF NMDAR antibody positive (%)	Gender		Age
						M	F	Mean ± SD
Busse et al. [28]	Typical dementia	AD classical	145	6 (4)	0	45	100	81
		VD	61	10 (16)	0	20	41	78
		FTD	34	3 (9)	0	14	20	77
	Control	Healthy	32	2 (6)	0	8	24	72
	Atypical dementia	Non-classical AD	11	11 (100)	0	5	6	81
Busse et al. [9]	Typical dementia	AD	46	4 (9)	–	16	30	80
		VD	26	4 (15)	–	10	16	74
		FTD	18	4 (22)	–	7	11	79
		DLB	11	1 (9)	–	6	5	76
	Control	Healthy	21	2 (10)	–	9	12	72
Coban et al. [30]	Typical dementia	AD classical	28	0	–	–	–	–
		FTD classical	6	0	–	–	–	–
	Control	Healthy	50	0	–	25	25	53 ± 16
	Atypical dementia	AD with atypical features	11	0	–	–	–	–
		FTD with atypical features	4	0	–	–	–	–
		DLB with atypical features	1	1 (100)	–	1	0	58
Doss et al. [31]	Typical dementia	AD	100	10 (10)	1 (1)	–	–	70
		VD	30	0	1 (3)	–	–	–
		FTD (inc PPA)	65	9 (14)	3 (5)	–	–	–
		PDD	25	5 (20)	0			
		LBD	11	2 (18)	0	–	–	–
		PSP	11	6 (55)	0	–	–	–
	Control	Healthy	47	2 (4)	0	–	–	67
	Atypical dementia	Unclassifiable dementia	20	12 (60)	4 (20)	–	–	–
Pruss et al. [19]	Typical dementia	AD	10	0	0	–	–	–
		FTD	9	1 (11)	1 (11)	–	–	–
		DLB	10	0	0	–	–	–
	Control	Healthy	75	0	0	–	–	–
	Atypical dementia	Progressive cognitive decline	23	6 (26)	2 (9)	8	15	–
Hopfner et al. [32]	Typical dementia	PDD	32	4 (13)	–	20	12	73 ± 6
	Control	Healthy	295	65 (22)	–	185	110	66 ± 6
Total typical dementia			678	69 (11)	6/511 (1)			
Total atypical dementia			70	30 (43)	6/54 (11)			

*AD* Alzheimer’s disease, *VD* vascular dementia, *FTD* frontotemporal dementia, *DLB* dementia with Lewy bodies, *PPA* primary progressive aphasia, *PDD* Parkinson’s disease dementia, *PSP* progressive supranuclear palsy

All studies recruited patients with clinical diagnoses of dementia; four of these confirmed diagnoses with clinical testing, including neuropsychological assessments and MRI [29–31, 33]. Three studies excluded patients with possibly confounding co-morbidities (e.g., infection, autoimmune disease, and cancer) [29–31]. One study recorded the prevalence of autoimmune disease and cancer in their cohort, but did not exclude these patients from the typical dementia group [32]. One study did not describe any confirmatory tests for dementia diagnoses or assessment for co-morbidities [19].

Four studies reported antibody prevalence in atypical dementia, i.e., patients characterised by symptoms or pathology that are not common to typical dementia syndromes [19, 29, 31, 32]. Two studies identified atypical cases through efforts to confirm dementia diagnoses among the recruited participants, and subsequently excluded these individuals from the typical dementia groups [29, 31]. Two other studies included targeted-recruitment of patients with atypical dementia [19, 32]. Atypical features in these cases included atypical onset or progression (presenile onset, rapid progression, subacute onset, atypical onset, partial regression, or plateau), CSF abnormalities (oligoclonal bands, pleocytosis, blood–brain-barrier dysfunction, or raised protein), and ‘immune challenge’ (autoimmune disease, cancer, or current infection). In the present analysis, these participants could not be classified under a typical dementia syndrome and were included as a heterogenous ‘atypical’ dementia group.

Not all studies described the recruitment process for healthy controls. Studies that did so excluded patients with neurodegenerative disorders [29], neurological or psychiatric disease [30], self-reported neuropsychiatric disease [33], or all of these [32]. One study specified exclusion of those with abnormalities in routine laboratory tests [31].

#### Antibody assays

Serum was collected from patients and healthy controls in all studies. Four of the six studies also collected CSF samples and three studies tested NMDAR antibodies in CSF. A few patients across studies were found to have positive CSF NMDAR antibodies, 12 in total (see Table 1) and only one study reported data with CSF and serum results matched for patients [19]. To increase statistical power, the present analysis only considered anti-NMDAR assays in serum. All studies used recombinant cell-based indirect immunofluorescence using fixed transfected HEK293 cells to detect anti-NMDAR antibodies, using non-transfected cells as negative controls. Non-NMDAR antigens were also investigated using various methods. Antigens included: GAD65, LGI1, CASPR2, AMPAR, and GABA_B_R [19, 31] and non-specified targets [33]. Four studies described blinded assays or analysis [29, 30, 32, 33]. Only NMDAR antibody assays were considered for meta-analysis; three studies reported results for IgG, IgA, and IgM [29, 30, 32], one reported IgA and IgM [33], one reported IgG only [31], and one reported IgA only [19].

#### Study-quality assessment

Study-quality ratings are provided in Table 2. Mean total quality score was 5.33 (out of a possible 12) and range was 0–8. Studies were awarded a point for sample size when a calculation to determine the appropriate sample size was performed. Where a sample size calculation was not described, we considered the overall prevalence of anti-neuronal autoantibodies in the population (approx. 5% in healthy and approx. 10% in disease groups [34]). We specified that five positive individuals in each group would permit a meaningful comparison, so group sizes of 100 healthy and 50 dementia were required to award a point. Studies that examined a total population (e.g., using health records) and included a large sample size were also awarded a point. Sample size was inadequate in two studies [19, 31]. Four of the six studies adequately defined and confirmed typical dementia diagnoses in cases [29, 30, 32, 33], but only one of these studies recruited a sample that was considered to be representative of the target population [33]. One other study recruited dementia cases that were likely representative, but the authors did not describe any attempt to confirm dementia diagnoses [32]. A few studies described recruitment of controls or commented on the extent to which these individuals were representative of the general population. Controls were representative of the general population in only one study [33].

**Table 2:** Assessment of risk of bias using modified version of the Newcastle–Ottawa Scale

References	Sample size (max 2: 1 cases, 1 controls)	Representativeness of cases (max 1)	Selection of dementia cases unbiased (max 1)	Adequate dementia definition confirmed (max 1)	Control group representative (max 1)	Control group unbiased (max 1)	Response rate reported and same in both groups (max 1)	Dementia and healthy matched (max 2)	Independent blinded assay (max 1)	All results reported	Total quality Score (max 12)
Busse et al. [29]	1	0	1	1	0	1	0	1	1	1	7
Busse et al. [9]	1	0	1	1	0	1	0	0	1	1	6
Coban et al. [31]	0	0	1	1	0	0	0	1	0	1	4
Doss et al. [32]	1	1	1	0	0	1	0	1	1	1	7
Hopfner et al. [33]	1	1	0	1	1	1	0	2	1	0	8
Pruss et al. [19]	0	0	0	0	0	0	0	0	0	0	0

Cases and controls were age matched in four studies [29, 31–33], and were also gender matched in one study [33]. Two studies failed to confirm that controls did not have exposures that might influence antibody prevalence (e.g., infection, autoimmune disease, immunosuppression, or cancer) [19, 31]. For antibody assays, all studies used an established method for determining seropositivity (i.e., cell-based assay) in cases and controls, but only four of the six studies described a blinded protocol, and serum dilutions were not reported in all studies. Two studies described assays for which no results were reported (for IgG NMDAR antibodies and for non-NMDAR antigens) [19, 33]. Response rate, which reflects the proportion of individuals who were invited to participate in the study and then agreed, was not reported for cases or controls in any study.

### Meta-analysis of frequency of IgG and IgA or IgM NMDAR antibodies

#### IgG NMDAR antibodies in dementia vs healthy age-matched controls

**Fig. 2:**
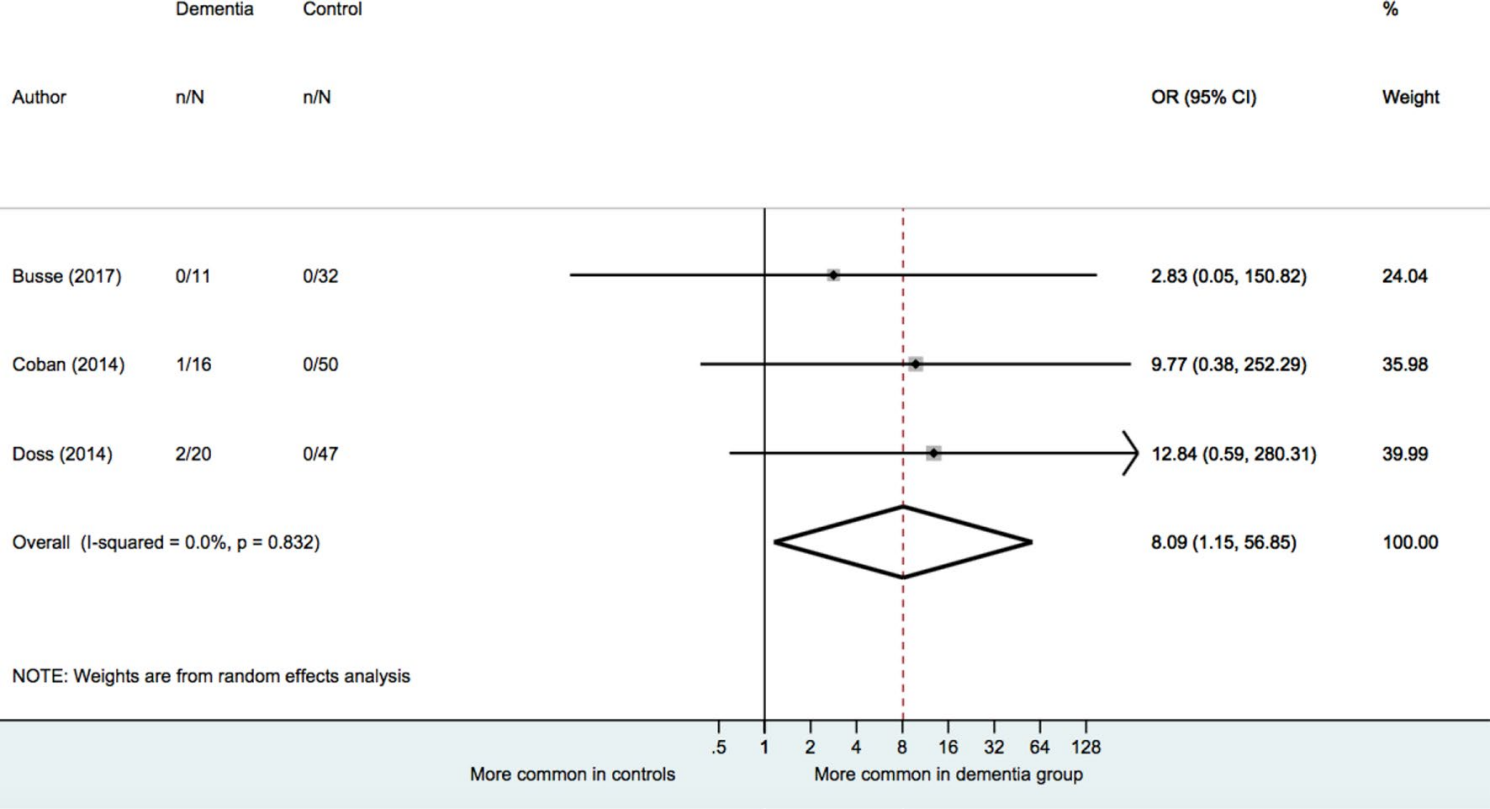
Meta-analysis of IgG NMDAR antibody in atypical dementia vs controls

Results of meta-analyses are provided in Table 3. When examining dementia of any type (typical and atypical groups combined), a random-effects model indicated no significant association between presence of IgG NMDAR antibodies and dementia status [OR = 1.05 (95% CI: 0.20 to 5.36), *p* = 0.957]. When analyses were performed separately for each dementia type, there was no significant association found between IgG NMDAR antibody presence and typical dementia status [OR = 0.72, (95% CI: 0.13 to 4.04), *p* = 0.710]. There was, however, an association found in the analysis restricted to atypical dementia types [OR = 8.09 (95% CI: 1.51 to 56.85), *p* = 0.036] (see Fig. 2). Heterogeneity estimates for all three analyses were small (*I*^2^ = 0.0%) and not statistically significant (*p* for Cochran's *Q* > 0.05).

**Table 3:** Meta-analyses comparing frequency of NMDAR antibody among dementia patients and healthy controls

	No. studies	Total *N* dementia/control	OR	95% CI	*P*	*P* for *Q*	*I*^2^ (%)
IgG NMDAR antibody							
All-case dementia	4	*664/150*	1.05	0.20–5.36	0.957	0.616	0.0
Typical dementia	4	*617/150*	0.72	0.13–4.04	0.710	0.799	0.0
Atypical dementia	3	47/129	**8.09**	**1.51–56.85**	**0.036**	0.832	0.0
IgM or IgA NMDAR antibody							
All-case dementia	5	698/470	1.93	(0.67–5.60)	0.227	0.053	57.3
Typical dementia	5	644/470	1.30	(0.58–2.90)	0.526	0.251	25.6
Atypical dementia	3	54/154	**42.48**	**(11.39–158.52)**	** < 0.001**	0.366	0.4

*OR* odds ratio, *CI* confidence interval, *Q* Cochran’s *Q* to detect statistically significant heterogeneity, *p < 0.05* indicates significant heterogeneity, *I*^2^ variation in OR attributable to heterogeneity

Bold indicates statistical significance at the 0.05 level (two-tailed)

#### IgA or IgM NMDAR antibodies in dementia vs healthy age-matched controls

**Fig. 3:**
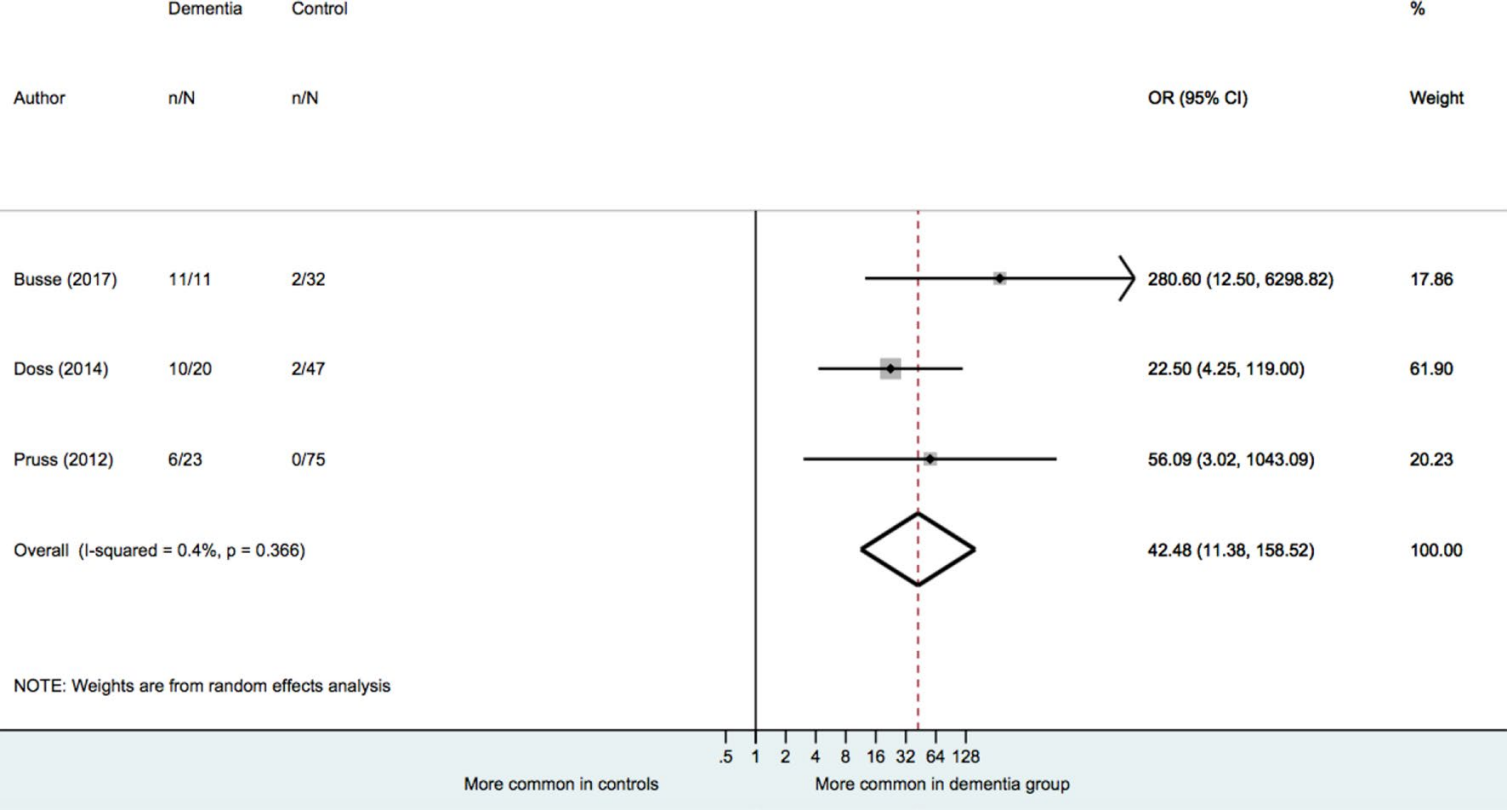
Meta-analysis of IgA or IgM NMDAR antibody in atypical dementia vs controls

A random-effects model also indicated no significant difference in the prevalence of IgA or IgM NMDAR antibodies between all dementia cases (typical and atypical) and healthy individuals, [OR = 1.93 (95% CI: 0.67 to 5.60), *p* = 0.227) or between typical dementia and healthy individuals [OR = 1.30 (95% CI: 0.58 to 2.90), *p* = 0.526] (see Table 3). However, there was a significantly higher prevalence of IgM or IgA NMDAR antibodies in atypical dementia when compared to healthy individuals [OR = 42.48 (95% CI: 11.39 to 158.52), *p* < 0.001] (see Fig. 3). The heterogeneity estimate for atypical dementia was low (*I*^2^ = 0.4%, *p* for Cochran’s *Q* = 0.366). Greater heterogeneity was seen for the all dementia and typical dementia analyses, but this did not reach statistical significance (*I*^2^ = 57.3%, *p* for Cochran’s *Q* = 0.053; *I*^2^ = 25.6%, *p* for Cochran’s *Q* = 0.251).

#### Meta-regression of study-quality score on effect size

Exploratory meta-regression analyses were performed to examine whether effect sizes were associated with study quality (NOS scores). No significant association was found in any of the six analyses.

## Discussion

This is the first systematic review and meta-analysis to investigate an association between NMDAR antibody seropositivity and primary dementia syndromes. Overall, no association was found between the presence of IgG or IgA/IgM serum NMDAR antibodies and dementia in typical dementias or when all dementia types were combined. However, when individuals with ‘atypical dementia’ were considered separately, both IgG and IgA/IgM NMDAR antibodies were significantly more common in patients than healthy controls.

We note a number of issues which necessitate caution when interpreting these results. Foremost, large confidence intervals are seen for all effect sizes and particularly for atypical dementia, due to the small-sample sizes of the individual studies. Furthermore, many of the included studies in this meta-analysis do not make explicit whether the ‘atypical’ criteria were defined prospectively. If this description was applied post hoc, the bias introduced would undermine the validity of the findings.

In addition, the description of atypical dementia was not universally applied across studies and it is possible that differences in this definition underlie much of the variation in antibody prevalence between the studies. Such definitions are prone to circularity (e.g., the presence of oligoclonal bands or serum NMDAR antibodies may lead a clinician to call this dementia syndrome ‘atypical’), although in most cases, there was additional evidence to suggest that the dementia syndrome was not typical.

There was a degree of heterogeneity in the description of ‘atypical’ dementia despite consensus across studies that these patients did not meet the full criteria for established dementia syndromes. Busse and colleagues defined ‘atypical’ dementia as patients without the imaging or CSF abnormalities characteristic of Alzheimer’s disease (i.e., minimal or no hippocampal atrophy or normal CSF Tau and B-amyloid) [29]. They also noted poorer prognostic outcomes and minimal symptomatic response to the usual cholinesterase inhibitor treatment in atypical dementia patients. Two studies established higher rates of CSF abnormalities within the ‘atypical’ group, with subacute onset and rapid symptomatic progression [31, 32]. Doss and colleagues also noted higher rates of so-called ‘immune challenges’ including autoimmunity amongst the ‘atypical’ group [32].

Notable similarities exist between the description of the ‘atypical’ dementias and NMDAR encephalitis itself, and so, the increased prevalence of NMDAR antibodies within this patient group is not unexpected [35]. Demographic differences are known to influence presentation in anti-NMDAR encephalitis, and thus, it seems possible that, in older adults, NMDAR antibodies could cause an attenuated encephalitic syndrome more closely resembling a neurodegenerative dementing process or an ‘autoimmune dementia’ [36, 37]. Indeed, numerous reports illustrate cases where antibody-mediated cognitive impairment is initially diagnosed as primary neurodegenerative dementias, and also evidence the positive outcomes when immunotherapy treatment is introduced [16–19, 31, 37]. This raises the possibility that autoimmune dementia could be considered where ‘atypical’ dementia, which does not meet the criteria for established dementia syndromes, is seen.

Previous studies that have sought to demonstrate the validity of the ‘autoimmune dementia’ concept have included patients due to clinical suspicion or confirmation of autoimmune encephalopathy rather than those chosen from an unselected dementia cohort [17], cementing the notion that autoimmune encephalitis can present with a dementia-predominant picture. Notably, in one such study, dementia was present in isolation in only 29% of an ‘autoimmune dementia’ cohort, with the occurrence of other neurological features occurring in the majority [17]. It is not clear whether or to what extent this category may overlap with the ‘atypical dementia’ category described in the present review, but the phenomenon of autoimmune dementia could be akin to the antibody-mediated syndromes described in autoimmune psychosis and autoimmune epilepsy [38, 39].

A causative link between NMDAR antibodies and atypical dementia has yet to be shown, but given the reversible nature of autoantibody-mediated processes, further investigation is warranted. We feel that it will be important for future studies to systematically and prospectively define the clinical features which indicate that NMDAR antibodies may contribute to the clinical symptoms, as well as address the described limitations.

In contrast, in established dementia syndromes such as Alzheimer’s dementia and Parkinson’s disease dementia where the prevalence of NMDAR antibodies was not found to be elevated, it seems less likely that they play a significant role in the pathogenesis. However, in Alzheimer’s disease and vascular dementia, NMDAR antibodies have been found to associate with psychiatric symptoms (particularly psychosis), suggesting that these antibodies could be biomarkers of neuropsychiatric symptoms in dementia even if they are not primarily pathogenic [9]. While Doss et al. did not replicate the association between NMDAR antibodies and neuropsychiatric symptoms in dementia, theirs was a cruder estimate looking at neuropsychiatric symptoms collectively without distinguishing psychotic symptoms from others [32]. Nonetheless, these findings also merit further investigation.

Consideration should also be given to the emerging notion that in some circumstances, NMDAR antibodies may have a role as endogenous NMDAR antagonists and hence have a protective function [40]: the possibility that in some cases dementia, serum NMDAR antibodies could represent an adaptive physiological response, potentially even ameliorating clinical severity, is challenging but requires systematic evaluation.

### IgG vs IgA/IgM NMDAR antibody

We had anticipated a distinction seen in the prevalence of IgG NMDAR antibodies and IgA or IgM NMDAR isotypes in dementia. IgG antibodies to the NR1 subunit of the NMDAR are recognised to be encephalitogenic, but the role of IgA and IgM NMDAR antibodies is less clear.

However, all NMDAR antibody isotypes have been found to have pathogenic potential, causing a reduction in the density of NMDARs with subsequent decreases in NMDAR-mediated currents [20]. Clinically, IgA NMDAR antibodies have been associated with a slowly progressive cognitive impairment, closer to a dementia phenotype but apparently reversible with immunotherapy [19]. Furthermore, non-IgG NMDAR antibodies have been implicated in the cognitive impairment seen in a range of disorders outside of encephalitis. IgA and IgM NMDAR antibodies are detected in cancer and in stroke at twice the frequency of IgG antibodies, with the cognitive impairment closely correlated to antibody titre in cancer [29, 41, 42].

While our results show greater prevalence of NMDAR antibodies in atypical dementia, no variation was seen across the antibody isotypes. It may be that any clinical variation is not picked up by the heterogeneous category of ‘atypical dementia’ and future studies should aim to define this clinical population more closely.

### Findings from systematic review

Although heterogeneity estimates were not statistically significant, our systematic review demonstrated substantial methodological variation between studies. The quality of included studies was moderate, with a mean rating of only 5.3 (12 representing the total maximum score). In particular, there was one outlier (which scored 0 overall) which contributed to the overall low mean score for quality [19]. Two of the studies did not match groups demographically, a major confounding factor given the prevalence of NMDAR antibodies is known to increase with age. Furthermore, no study was adequately powered to detect differences in frequency of NMDAR antibodies with most studies only reaching adequate sample size for either patients or controls. Only one study ensured representativeness of cases and controls which limits the extent to which sampling bias can be assessed. While there was no measure that all studies universally achieved, most studies had an independent blinded assay for the measurement of NMDAR antibodies.

### Limitations

While this is the first systematic review and meta-analysis to investigate the prevalence of neuronal autoantibodies in dementia syndromes, a number of issues limit the scope of conclusions drawn.

The strict methodology and small number of studies available for meta-analysis increase the risk of type II error. Our finding of no association between serum NMDAR antibodies and dementia in either typical dementias or all dementia types combined could potentially represent a false-negative result. Due to the small numbers of studies available, analyses were only possible for NMDAR antibodies. One study was identified in the systematic review aiming to identify the prevalence of AMPAR antibodies, but in the absence of any others, it was excluded. However, it could be that the paucity of studies is explained by the relatively low prevalence of these autoantibodies in dementia syndromes, as is alluded to by Hopfner and colleagues who did not include analyses for other tested antibodies due to their low prevalence [33].

We only included serum NMDAR antibodies in our meta-analysis. However, increasingly, CSF autoantibodies are felt to be required for definitive diagnosis of most autoimmune encephalitis subtypes. This requirement reflects the problem of nonspecificity of many serum-only autoantibodies, i.e., the need to avoid false-positive diagnoses in the context of elevated rates of such antibodies detected in healthy populations or disease controls (where their significance is uncertain), as well as the notion that intrathecal antibody synthesis is necessary (and probably sufficient) for encephalitis pathogenesis [43]. Only three of the included studies tested NMDAR antibodies in the CSF of patients and few had a positive NMDAR antibody (12 positives overall; 2% NMDAR antibody positive of CSF tested patients with dementia vs 13% of NMDAR antibody seropositive patients with dementia). However, given the smaller patient numbers undergoing NMDAR antibody CSF testing, it is difficult to interpret this finding further. Future studies should look to simultaneously test both serum and CSF for NMDAR antibodies. Furthermore, future studies should incorporate paraclinical testing including EEG and MRI, since abnormalities in these modalities may provide further support for an antibody’s pathogenicity.

The small number of studies also prevented more robust analyses comparing effect sizes across individual groups. Due to insufficient numbers, we were not able to separate out dementia subtypes and instead grouped patients into ‘typical’ or ‘atypical’ syndromes. Thus, we cannot be specific as to any differences in NMDAR antibody frequencies between dementia subtypes and given the different aetiologies and clinical presentations underpinning respective dementia subtypes, this is a major limitation. Furthermore, due to the small sample-sizes tested, large confidence intervals are seen for the autoantibody frequencies within atypical dementias.

### Conclusions and recommendations

We did not find evidence to suggest NMDAR antibodies are more common in AD or other dementia-related diseases than in healthy controls. However, both IgG and IgM or IgA NMDAR serum antibodies were found to be more common in ‘atypical’ dementia vs healthy controls. Due to the methodological issues described, we advise caution interpreting this result and suggest that further investigation is warranted. Only three of the included studies examined patients with atypical dementia and in each, due to the small-sample sizes, the confidence intervals were extremely wide.

This meta-analysis supports the need for additional, methodologically robust studies to investigate an association between NMDAR antibodies and dementia. To improve study quality and reduce the risk of bias, we recommend future studies include larger sample sizes, measure antibodies in CSF and serum, aim to apply a consistent, prospective definition to patients with ‘atypical’ dementia, and endeavour to match groups on confounders such as age and gender to allow more robust analyses. In particular, future research would benefit from a larger, prospective study to determine the prevalence of NMDAR antibodies in ‘atypical’ dementia.

## Abbreviations

NMDAR: *N*-Methyl-d-aspartate receptor
LGI1: Leucine-rich glioma inactivated 1
CASPR2: Contactin-associated protein 2
AMPAR: α-Amino-3-hydroxy-5-methyl-4-isoxazolepropionic acid receptor
GABA: Gamma-aminobutyric acid
GAD: Glutamic acid decarboxylase
